# NaBiF_4_-based hollow upconversion nanoparticles for temperature sensing

**DOI:** 10.1038/s41377-022-00954-x

**Published:** 2022-08-25

**Authors:** Lining Sun

**Affiliations:** Department of Chemistry, College of Sciences, Shanghai University, 200444 Shanghai, China

**Keywords:** Nanoparticles, Optical techniques

## Abstract

Hollow upconversion nanoparticles with tunable central cavity size can be used as self-referenced luminescent thermometers over a wide temperature range.

Temperature represents the degree of cold and hot of an object and is a basic physical parameter in scientific and industrial applications^1^. Temperature measurement method that does not require additional calibration during the measurement process, that is, self-referenced thermometry, shows great application prospects^2^. Self-referenced thermometers based on upconversion luminescence (UCL) have obvious advantages in temperature detection because of high sensitivity, non-contact, and tolerance to extreme conditions^3^.
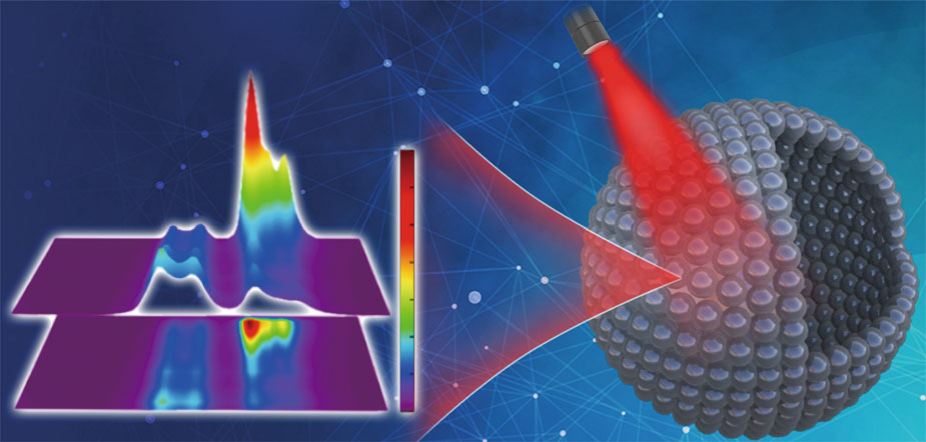


The phenomenon of UCL was discovered in the 1960s^4,5^, but it was not until recent decades that lanthanide-doped upconversion nanoparticles (UCNPs) developed rapidly with the advent of nanotechnology^6,7^. UCNPs have been extensively investigated in the field of temperature sensing due to the large anti-Stokes shifts, sharp-band emissions, long lifetimes, low toxicity, weak autofluorescence, and adequate thermal stability^8,9^.

With the deepening of research, this field has reached a mature level. Researchers can now synthesize a variety of UCNPs with the desired size, morphology, structure, and functions^10–12^. Compared with other morphological materials, there are relatively few studies on hollow UCNPs and their optical applications, mainly because of the difficulty in constructing hollow structures by general methods. However, hollow structure nanoparticles have the characteristics of large internal surface area and high surface permeability^13^, so that they are expected to have high light collection efficiency, which is conducive to obtaining excellent luminescence properties. The structural advantages of hollow UCNPs are well worth further studying.

Now, writing in this issue of *Light: Science & Applications*, Prof. Hongjie Zhang and colleagues at the State Key Laboratory of Rare Earth Resource Utilization, Changchun Institute of Applied Chemistry, Chinese Academy of Sciences, China report a one-step template-free method for the synthesis of NaBiF_4_:Yb,Er hollow UCNPs^14^. Moreover, they achieved controllable tuning of the cavity size in the nanoparticles. NaBiF_4_:Yb,Er hollow nanoparticles exhibit excellent luminescence properties under 980 nm near-infrared irradiation due to the advantages of the hollow structure. The authors also proposed the possible formation mechanism of hollow structure, which will provide guidance for future research on hollow UCNPs.

With the presented work, Prof. Hongjie Zhang and co-authors demonstrate that the NaBiF_4_:Yb,Er hollow nanoparticles could be employed as self-referenced ratiometric luminescent thermometers. Furthermore, the high stability of these nanoparticles ensures their sensing ability over a wide temperature range. In addition, the excitation wavelength of the self-referenced thermometer developed by Prof. Hongjie Zhang et al. locates in the near-infrared region, which makes them have great potential for temperature sensing in the biological field in the future.

Hence, the presented work provides a new avenue for the synthesis of hollow nanoparticles and exploring their optical applications. Nevertheless, further optimization of materials will be required to improve luminescence performance and sensor sensitivity. In the future, it is possible that hollow UCNPs can not only serve as carriers to deliver drugs in vivo but also monitor the entire delivery process and real-time temperature, which will expand the application scenarios of hollow UCNPs.
